# X-Rays and Other Tests for Death

**Published:** 1908-03-28

**Authors:** Jas. R. Williamson

**Affiliations:** 100 Chedington Road. Upper Edmonton, London, N.


### EDITOR'S LETTER-BOX.

### X-RAYS AND OTHER TESTS FOR DEATH.

Sir,—With reference to the able and instructive article on the signs of death in your valuable journal of March 21, may I be permitted to remark that while the *x*-rays may be useful in the diagnosis of normal cases of life and death, what is wanted for the prevention of premature burial is a ready scientific test, capable of universal application, which would with absolute certainty distinguish between trance, catalepsy, and other forms of suspended animation and real death? It cannot be too strongly urged and widely known that the only trustworthy proof of death is the advent of putrefactive decomposition (the late Sir Henry Thompson, F.R.C.S., etc.). The Rev. Dr. G. B. Geniesse, of Rome, who has translated and published in various languages a treatise entitled "La Mort Réelle et la Mort Apparente," in a letter to the present writer says: "Very likely you have heard of the new sign of death proposed by Icard in his book 'Le signe de la Mort réelle en l'absence du Médecin de la réaction sulphytrique.' I was already acquainted with that sign secretly. The proofs of Icard do not convince me. So many others have proposed signs as certain, and it was afterwards proved that they were mistaken. We must fear very much the action of auto-suggestion in these matters. A friend of mine communicated a case narrated by Senator (Berliner Klinische Wochenische Tobey, p. 214, 1868), which destroys the doctrine of Icard. He had found that case by accident in another book abridged from the Berlin journal. I have written for the complete article. It is necessary that many other physicians try the new sign of Icard on all kinds of sick and dying people, which was not sufficiently done by Icard. Besides, even supposing that sign is certain, all difficulties are not then removed; the paper becomes black a little before the appearance of the green coloration of the abdomen, but we know that this coloration in many cases only shows itself after two, three, four, five, eight, and ten days, and at present laws and customs do not permit to await such a long time before the burial. The sign being proved, difficulties are diminished, but not destroyed." Dr. Icard's fluorescin test was made public in 1897, yet I am unaware that anyone has yet received the 25,000 francs offered by the French Academy of Medicine for the discovery of a simple and certain means of recognising whether a body is dead or only apparently so. I should be pleased to send a copy of "The Signs and Proofs of Death," prepared by the late Sir B. W. Richardson, M.D., F.R.S., on receipt of a stamped addressed envelope. If medical practitioners were to adopt these valuable diagnostic signs of death, and apply them in all cases, the existing dangers of premature burial would be greatly, if not entirely, removed.

I am sir, your obedient servant,

Jas. R. Williamson.

100 Chedington Road, Upper Edmonton,

